# Supplementary material for: A few Ascomycota taxa dominate soil fungal communities worldwide
Source: Nat Commun. 2019 May 30;10:2369. doi: 10.1038/s41467-019-10373-z (PMC6542806; doi:10.1038/s41467-019-10373-z)
Supplement: Supplementary file 1 — Supplementary Information [file 41467_2019_10373_MOESM1_ESM.pdf]

Supplementary Information

Egidi et al. *A few Ascomycota taxa dominate soil fungal communities worldwide*

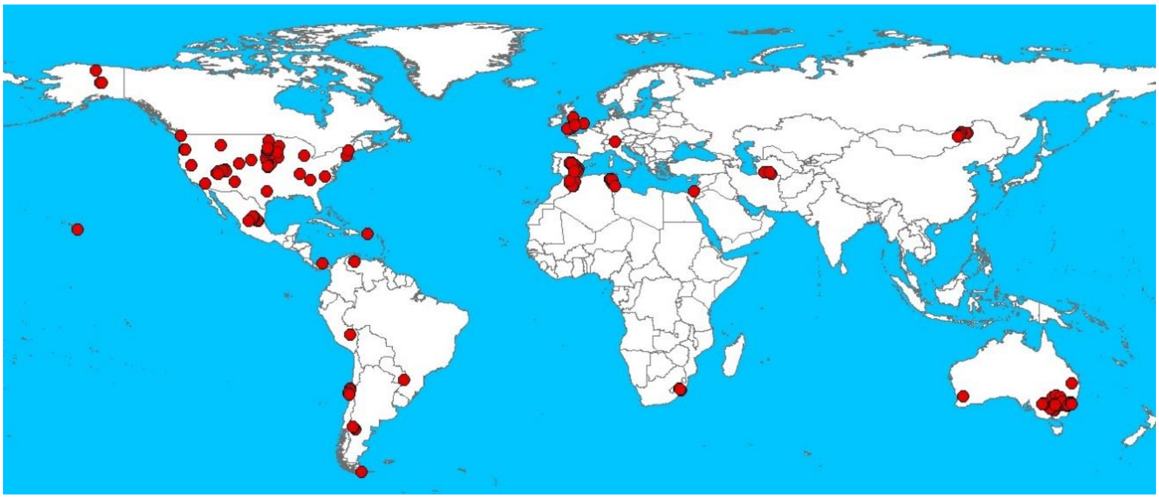

Supplementary Figure 1. Location of the sites surveyed in this study.

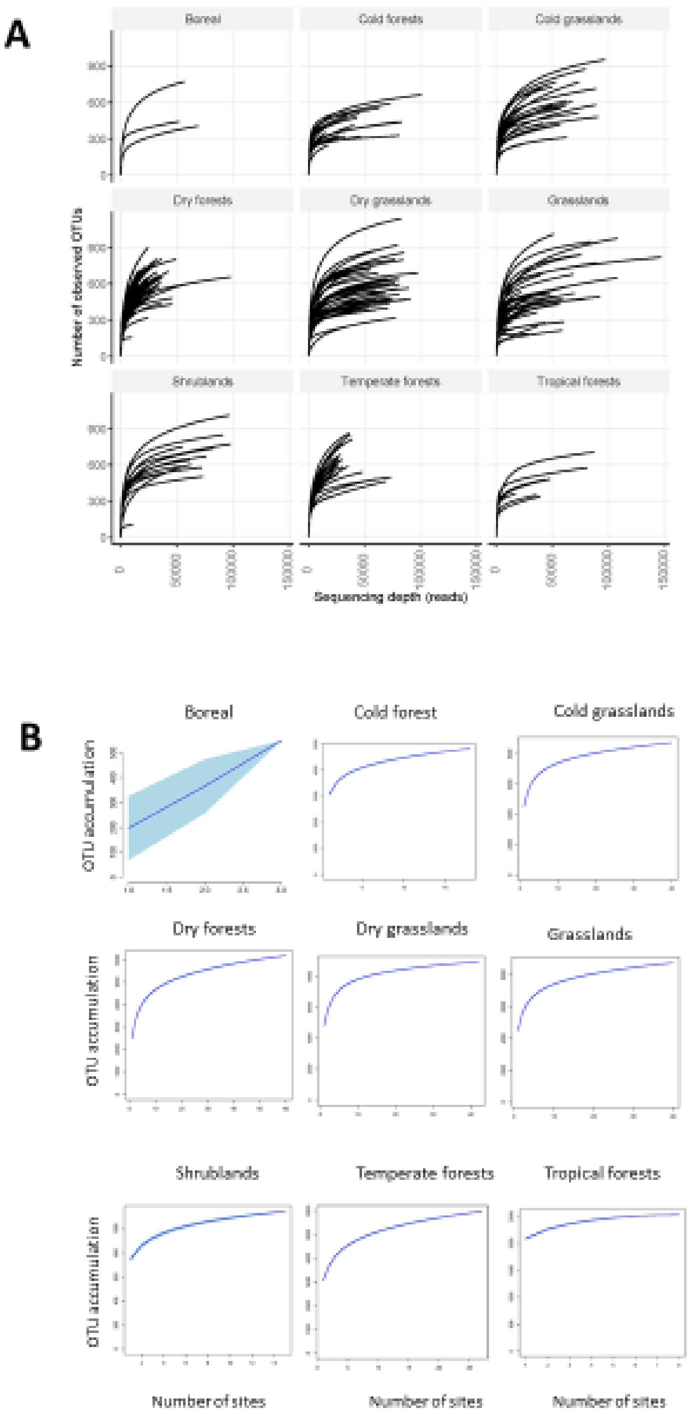

Supplementary Figure 2. Rarefaction (A) and species accumulation (B) curves for the samples analysed in this study. The rarefaction curves were obtained using the amp_rarecurve function in the package ampvis2 (https://madsalbertsen.github.io/ampvis2/index.html), while the species accumulation curves were obtained using the specaccum function (method=”rarefaction”) in the package vegan[1]. Shaded areas indicated 95% confidence intervals.

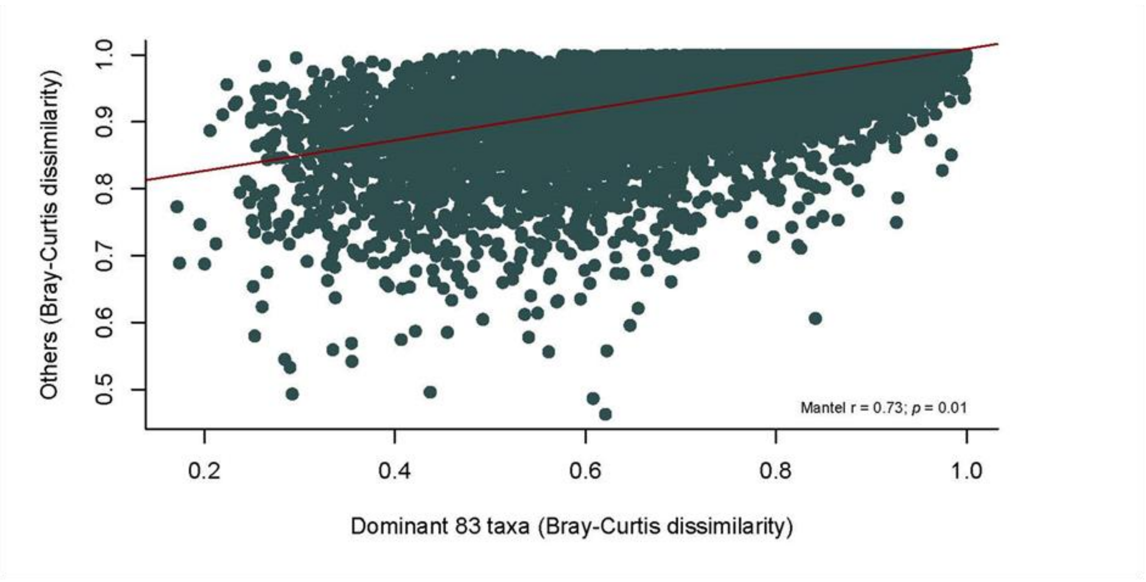

Supplementary Figure 3. Relationship between beta diversity (community dissimilarity) based on Bray-Curtis distance for the dominant (84 phylotypes) and the remaining 24,053 fungal phylotypes.

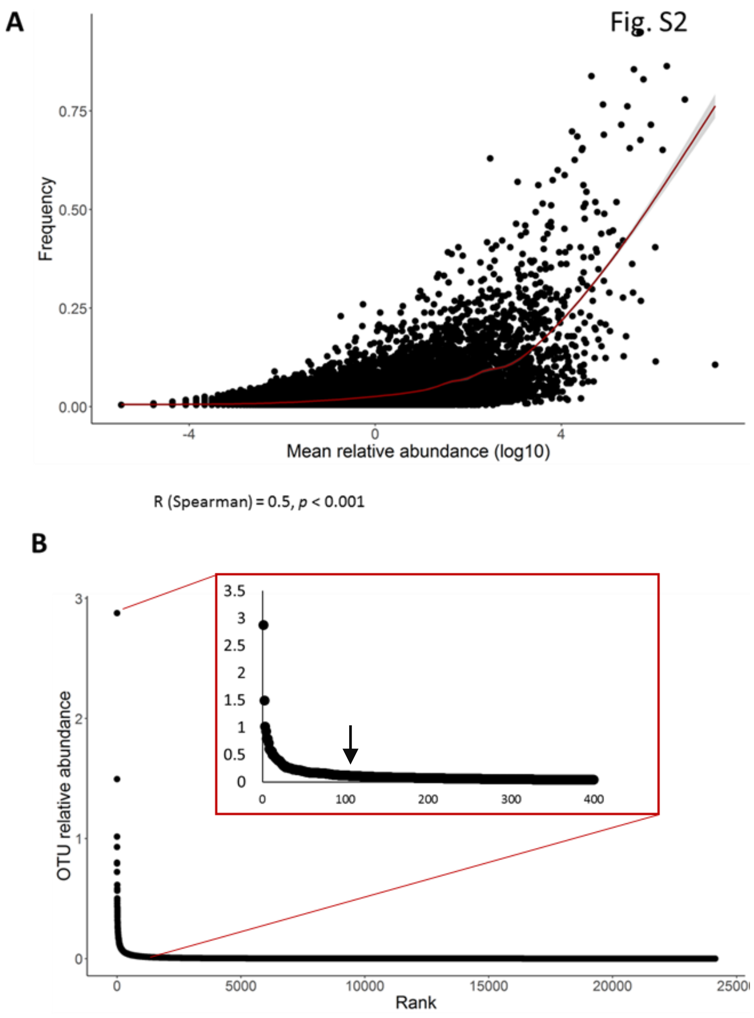

Supplementary Figure 4. Relationship between phylotype relative abundance and number of sites occupied (A) and rank abundance curve for fungal ITS sequence data, with the phylotypes ordered on the x-axis from most to least abundant (B). A close up of the top 400 taxa is shown in the inset. Only 130 OTUs had relative abundance >0.1% (indicated by an arrow in the inset).

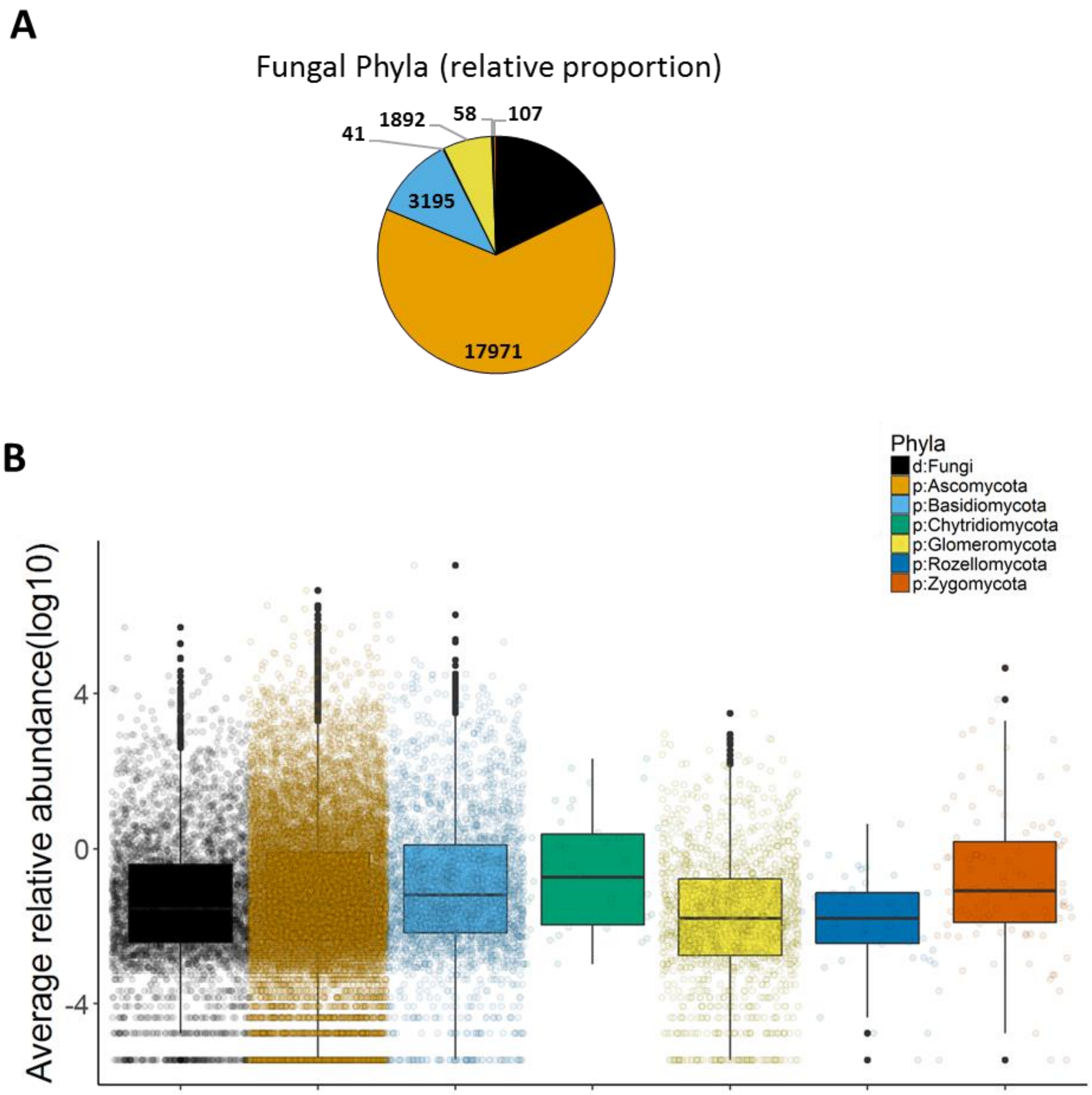

Supplementary Figure 5. (A) Pie chart indicating the relative abundance of the main fungal phyla retrieved at the global level. The number of phylotypes are reported for each phylum. (B) Boxplot comparing the average relative abundance (logarithmic scale) of each phylum.

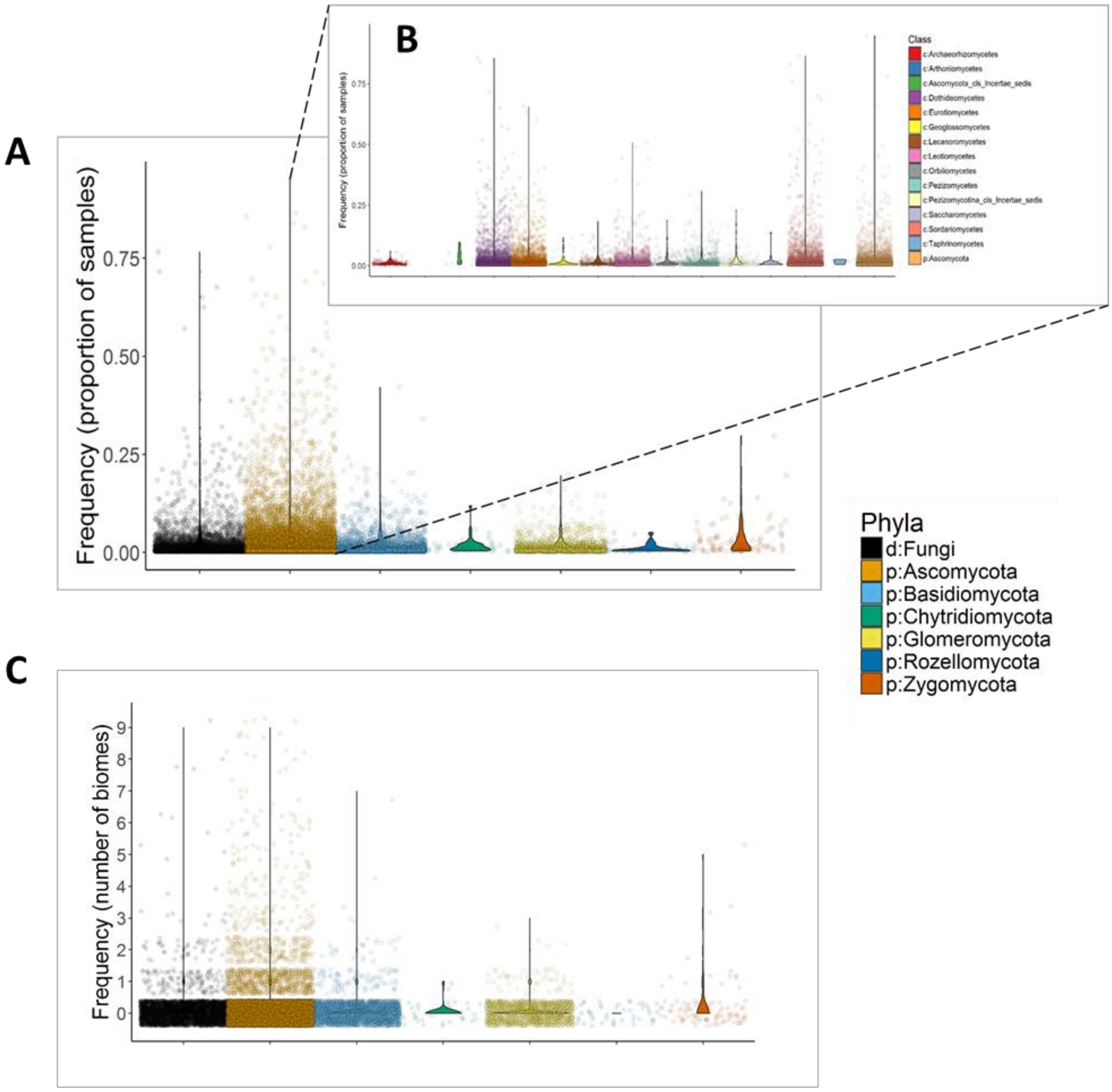

Supplementary Figure 6. Violin plots showing the number of samples in which each phylotype was observed, grouped by phylum (A), the number of samples in which each phylotype assigned to the phylum Ascomycota was observed, grouped by class (B) and the number of biomes in which each phylotype was found to be dominant (i.e., occurring in at least 30% of the samples from a given biome), grouped by phylum (C).

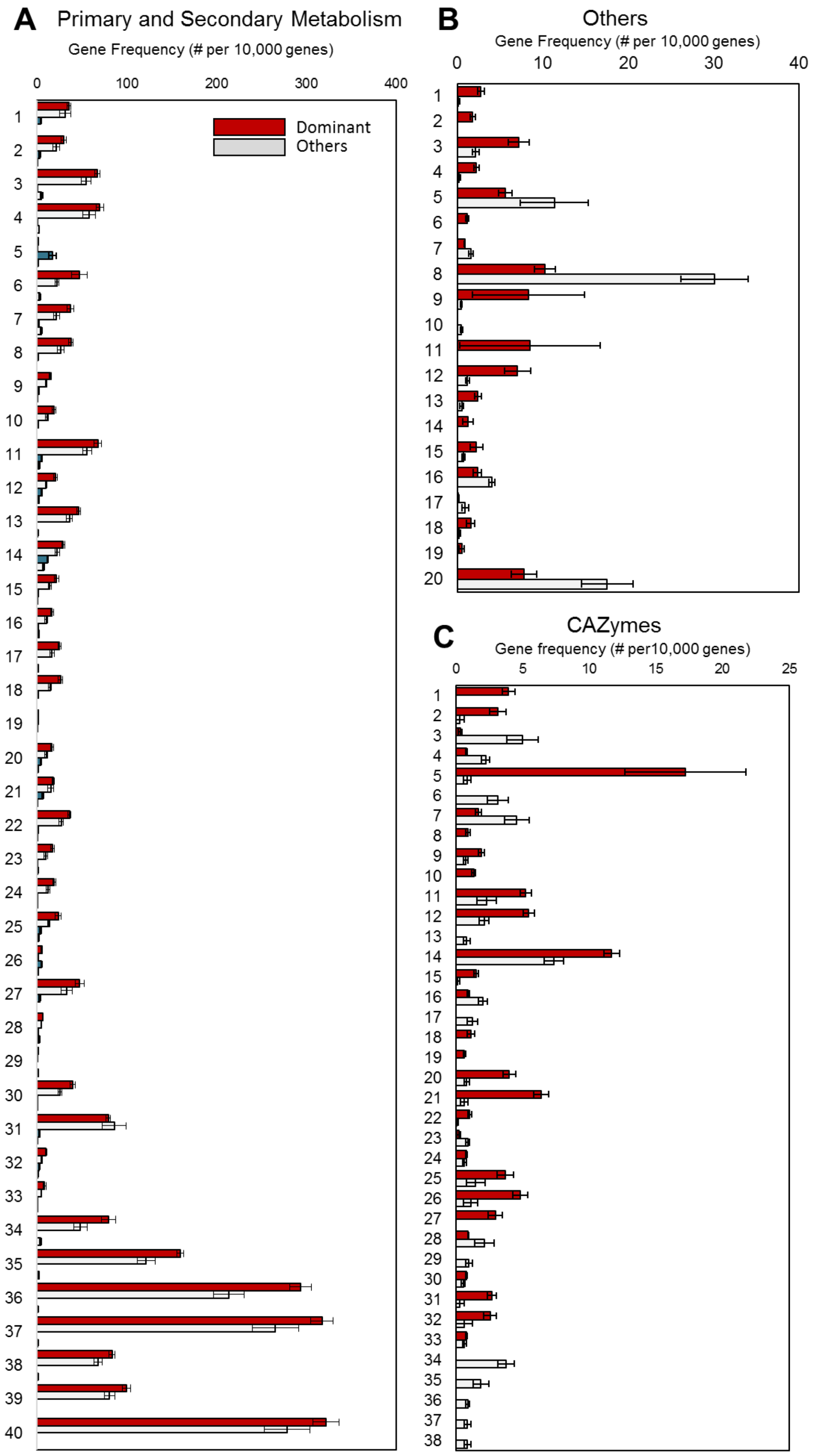

A
Primary and Secondary Metabolism
Gene Frequency (# per 10,000 genes)
0
100
200
300
400
Dominant
Others
1
2
3
4
5
6
7
8
9
10
11
12
13
14
15
16
17
18
19
20
21
22
23
24
25
26
27
28
29
30
31
32
33
34
35
36
37
38
39
40
B
Others
Gene Frequency (# per 10,000 genes)
0
10
20
30
40
1
2
3
4
5
6
7
8
9
10
11
12
13
14
15
16
17
18
19
20
C
CAZymes
Gene frequency (# per10,000 genes)
0
5
10
15
20
25
1
2
3
4
5
6
7
8
9
10
11
12
13
14
15
16
17
18
19
20
21
22
23
24
25
26
27
28
29
30
31
32
33
34
35
36
37
38

Dominant Ascomycota/Non-Dominant Basidiomycota

Fig.	#	Classification	ID	Function	Mean Decrease Accuracy	p value	p.value (one-way anova)
A	1	KEGG		Anthocyanin biosynthesis	2.34	0.04	*
	2	KEGG		Betalain biosynthesis	2.43	0.03	*
	3	KEGG		Biosynthesis of alkaloids derived from terpenoid and polyketide	2.73	0.03	*
	4	KEGG		Biosynthesis of unsaturated fatty acids	3.41	0.02	*
	5	KEGG		Biosynthesis of vancomycin group antibiotics	3.06	0.02	*
	6	KEGG		Caprolactam degradation	2.36	0.04	*
	7	KEGG		Cyanoamino acid metabolism	4.07	0.01	*
	8	KEGG		Fatty acid biosynthesis	3.44	0.02	*
	9	KEGG		Fatty acid elongation in mitochondria	4.39	0.01	*
	10	KEGG		Flavone and flavonol biosynthesis	2.17	0.04	*
	11	KEGG		Fructose and mannose metabolism	3.96	0	*
	12	KEGG		Geraniol degradation	2.69	0.03	*
	13	KEGG		Glycerophospholipid metabolism	2.56	0.03	*
	14	KEGG		Glycosphingolipid biosynthesis ganglio series	2.48	0.03	*
	15	KEGG		Glycosphingolipid biosynthesis globo series	5	0	*
	16	KEGG		Glycosphingolipid biosynthesis lacto and neolacto series	4.26	0.01	*
	17	KEGG		Glycosylphosphatidylinositol GPI anchor biosynthesis	2.85	0.02	*
	18	KEGG		High mannose type N glycan biosynthesis	2.49	0.03	*
	19	KEGG		Indole alkaloid biosynthesis	5.76	0	*
	20	KEGG		Keratan sulfate biosynthesis	2.75	0.02	*
	21	KEGG		Methane metabolism	3.41	0.02	*
	22	KEGG		N Glycan biosynthesis	4.54	0.01	*
	23	KEGG		O Glycan biosynthesis	4.81	0	*
	24	KEGG		O Mannosyl glycan biosynthesis	3.78	0.01	*
	25	KEGG		Pantothenate and CoA biosynthesis	5.87	0	*
	26	KEGG		Penicillin and cephalosporin biosynthesis	2.52	0.02	*
	27	KEGG		Phenylalanine metabolism	2.81	0.03	*
	28	KEGG		Phosphonate and phosphinate metabolism	5.07	0	*
	29	KEGG		Polyketide sugar unit biosynthesis	5.28	0.01	*
	30	KEGG		Propanoate metabolism	2.24	0.04	*
	31	KEGG		Sphingolipid metabolism	3.91	0.01	
	32	KEGG		Streptomycin biosynthesis	5.2	0.01	*
	33	KEGG		Synthesis and degradation of ketone bodies	2.32	0.05	*
	34	KEGG		Tyrosine metabolism	4.63	0	*
	35	KOG		Inorganic ion transport and metabolism	12.81	0	*
	36	KOG		Amino acid transport and metabolism	12.47	0	*
	37	KOG		Carbohydrate transport and metabolism	8.25	0.01	*
	38	KOG		Nucleotide transport and metabolism	6	0.02	*
	39	KOG		Coenzyme transport and metabolism	5.9	0.03	*
	40	KOG		Energy production and conversion	5.41	0.03	*
B	1	Nutrients	*Phosphate transporter/IPR001204*	P immobilization	23.11	0	*
	2	Nutrients	*Amino acid permease/IPR004762*	N immobilization	21.72	0	*
	3	SM	*PKS*	Stress resistance	19.42	0	
	4	SM	*PKS-LIKE*	Stress resistance	15.46	0	
	5	SM	*MRPS-LIKE*	Stress resistance	10.23	0.02	
	6	Stress	*β1,3-glucan synthase/GO 0006582*	C deposition	27.94	0	*
	7	Stress	*Melanin/GO 0000148*	C deposition	19.09	0.01	*
	8	MEROPS	*AA*	Peptidase	10.84	0	*
	9	MEROPS	*JC*	Peptidase	9.36	0	*
	10	MEROPS	*MM*	Peptidase	6.14	0	
	11	MEROPS	*AF*	Peptidase	2.57	0	
	12	MEROPS	*AD*	Peptidase	7.8	0	
	13	MEROPS	*MC*	Peptidase	7.31	0.01	*
	14	MEROPS	*AC*	Peptidase	5.63	0.01	
	15	MEROPS	*ME*	Peptidase	6.38	0.02	
	16	MEROPS	*MP*	Peptidase	7.12	0.02	*
	17	MEROPS	*MF*	Peptidase	4.52	0.03	
	18	MEROPS	*MERCO:U52 UPW*	Peptidase	4.19	0.04	*
	19	MEROPS	*CO*	Peptidase	2.54	0.05	*
	20	MEROPS	*MERCO:C108 00*	Peptidase	3	0.05	*
C	1	CAZy	*Auxiliary Activity Family 1 Subf 3*	Stimulation of lignocellulose degradation	6.27	0	*
	2	CAZy	*Auxiliary Activity Family 11*	Stimulation of lignocellulose degradation	3.88	0.01	*
	3	CAZy	*Auxiliary Activity Family 5 Subf 1*	Stimulation of lignocellulose degradation	5.14	0	*
	4	CAZy	*Auxiliary Activity Family 6*	Stimulation of lignocellulose degradation	4.15	0	*
	5	CAZy	*Carbohydrate Binding Module Family 18*	Catalytic efficiency enhancing	3.54	0.02	*
	6	CAZy	*Carbohydrate Binding Module Family 5*	Catalytic efficiency enhancing	3.63	0	*
	7	CAZy	*Carbohydrate Esterase Family 16*		2.78	0.04	*
	8	CAZy	*Glycoside Hydrolase Family 114*	Degradation of complex sugars	2.42	0.03	*
	9	CAZy	*Glycoside Hydrolase Family 125*	Degradation of complex sugars	3.56	0.01	*
	10	CAZy	*Glycoside Hydrolase Family 132*	Degradation of complex sugars	6.97	0	*
	11	CAZy	*Glycoside Hydrolase Family 17*	Degradation of complex sugars	3.77	0.01	*
	12	CAZy	*Glycoside Hydrolase Family 2*	Degradation of complex sugars	2.51	0.04	*
	13	CAZy	*Glycoside Hydrolase Family 23*	Degradation of complex sugars	4.12	0	
	14	CAZy	*Glycoside Hydrolase Family 3*	Degradation of complex sugars	2.3	0.05	*
	15	CAZy	*Glycoside Hydrolase Family 36*	Degradation of complex sugars	3.72	0.01	*
	16	CAZy	*Glycoside Hydrolase Family 5 Subf 12*	Degradation of complex sugars	2.76	0.04	*
	17	CAZy	*Glycoside Hydrolase Family 5 Subf 50*	Degradation of complex sugars	3.5	0	*
	18	CAZy	*Glycoside Hydrolase Family 64*	Degradation of complex sugars	3.12	0.01	*
	19	CAZy	*Glycoside Hydrolase Family 67*	Degradation of complex sugars	2.9	0.01	
	20	CAZy	*Glycoside Hydrolase Family 72*	Degradation of complex sugars	3.47	0.01	*
	21	CAZy	*Glycoside Hydrolase Family 76*	Degradation of complex sugars	5.46	0	*
	22	CAZy	*Glycoside Hydrolase Family 81*	Degradation of complex sugars	5.09	0	*
	23	CAZy	*Glycoside Hydrolase Family 85*	Degradation of complex sugars	2.32	0.05	
	24	CAZy	*GlycosylTransferase Family 21*	Fungal polysaccharide synthesis	2.9	0.02	
	25	CAZy	*GlycosylTransferase Family 31*	Fungal polysaccharide synthesis	2.47	0.03	*
	26	CAZy	*GlycosylTransferase Family 32*	Fungal polysaccharide synthesis	2.79	0.04	*
	27	CAZy	*GlycosylTransferase Family 34*	Fungal polysaccharide synthesis	5.58	0	*
	28	CAZy	*GlycosylTransferase Family 48*	Fungal polysaccharide synthesis	4.24	0	*
	29	CAZy	*GlycosylTransferase Family 49*	Fungal polysaccharide synthesis	3.44	0.01	*
	30	CAZy	*GlycosylTransferase Family 50*	Fungal polysaccharide synthesis	2.19	0.05	*
	31	CAZy	*GlycosylTransferase Family 62*	Fungal polysaccharide synthesis	3.87	0	*
	32	CAZy	*GlycosylTransferase Family 71*	Fungal polysaccharide synthesis	3.71	0.01	*
	33	CAZy	*GlycosylTransferase Family 76*	Fungal polysaccharide synthesis	2.37	0.03	*
	34	CAZy	*PolysaccharideLyaseFamily 14*	Degradation of Polysaccharide	5.95	0	*
	35	CAZy	*PolysaccharideLyaseFamily 14 Subf 4*	Degradation of Polysaccharide	3.74	0	*
	36	CAZy	*PolysaccharideLyaseFamily 14 Subf 5*	Degradation of Polysaccharide	6.43	0	*
	37	CAZy	*PolysaccharideLyaseFamily 8*	Degradation of Polysaccharide	2.36	0.02	*
-	38	CAZy	*PolysaccharideLyaseFamily 8 Subf 4*	Degradation of Polysaccharide	1.99	0.03	*

Supplementary Figure 7. Frequencies of genes selected from Random Forest (RF) analyses of genomes matching dominant Ascomycota phylotypes (n=13) and genomes representing phylotypes assigned to the non-dominant Basidiomycota (n=9). The asterisk highlights those genes that were significantly different in relative frequency (average ± SE), calculated by one-way anova, $p < 0.05$.

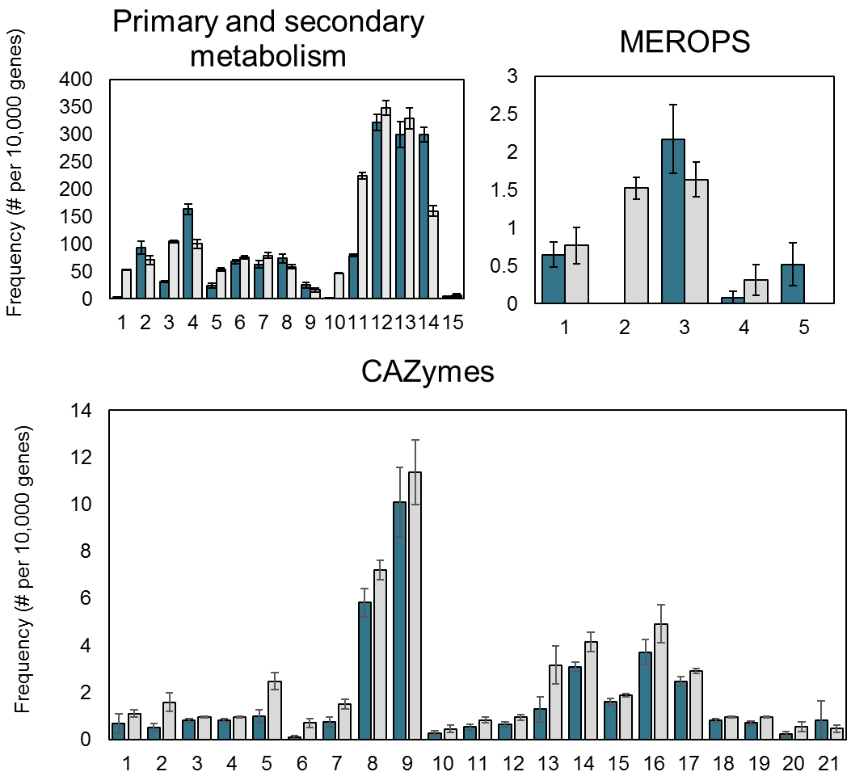

Dominant Ascomycota/Not Dominant Ascomycota

Fig.	#	Classification	ID	Function	Mean Decrease Accuracy	p value	p value (one-way anova)
A	1	KEGG	*Phosphate transporter/IPR001204*	Phosphate transporter	11.76	0.03	*
	2	KEGG		Benzoate degradation via CoA ligation	2.77	0.03	
	3	KEGG		Benzoxazinoid biosynthesis	3.90	0.01	
	4	KEGG		Fluorene degradation	2.46	0.04	
	5	KEGG		Fructose and mannose metabolism	4.42	0.01	
	6	KEGG		Methylnaphthalene degradation	3.33	0.02	
	7	KEGG		Naphthalene and anthracene degradation	3.52	0.01	
	8	KEGG		Primary bile acid biosynthesis	6.11	0	*
	9	KEGG		Sesquiterpenoid biosynthesis	3.05	0.01	
	10	KEGG		Sphingolipid metabolism	2.86	0.04	
	11	KOG		Energy production and conversion	6.15	0.02	*
	12	KOG		Secondary metabolites biosynthesis transport and catabolism	7.61	0.03	
	13	KOG		Translation ribosomal structure and biogenesis	5.96	0.02	
	14	KEGG		Biosynthesis of plant hormones	2.18	0.05	
	15	CARD		Antibiotic target replacement	10.93	0.015	*
B	1	MEROPS	*CF*	Peptidase	5.67	0.02	
	2	MEROPS	*PD*	Peptidase	5.53	0.01	
	3	MEROPS	*SF*	Peptidase	4.37	0.04	
	4	MEROPS	*MT*	Peptidase	3.59	0.05	
	5	MEROPS	*CO*	Peptidase	3.07	0.03	
C	1	CAZy	*Glycoside Hydrolase Family 127*	Degradation of complex sugars	4.41	0.01	
	2	CAZy	*Glycoside Hydrolase Family 13 Subf 22*	Degradation of complex sugars	2.22	0.04	*
	3	CAZy	*Glycoside Hydrolase Family 13 Subf 25*	Degradation of complex sugars	3.22	0.01	
	4	CAZy	*Glycoside Hydrolase Family 133*	Degradation of complex sugars	2.07	0.04	
	5	CAZy	*Glycoside Hydrolase Family 30*	Degradation of complex sugars	4.37	0.01	*
	6	CAZy	*Glycoside Hydrolase Family 30 Subf 3*	Degradation of complex sugars	2.53	0.03	*
	7	CAZy	*Glycoside Hydrolase Family 30 Subf 7*	Degradation of complex sugars	3.35	0.02	
	8	CAZy	*Glycoside Hydrolase Family 31*	Degradation of complex sugars	2.77	0.04	
	9	CAZy	*Glycoside Hydrolase Family 43*	Degradation of complex sugars	2.50	0.03	
	10	CAZy	*Glycoside Hydrolase Family 5 Subf 24*	Degradation of complex sugars	2.86	0.03	
	11	CAZy	*Glycoside Hydrolase Family 53*	Degradation of complex sugars	2.02	0.05	
	12	CAZy	*Glycoside Hydrolase Family 67*	Degradation of complex sugars	3.74	0.01	
	13	CAZy	*Glycoside Hydrolase Family 71*	Degradation of complex sugars	2.98	0.02	
	14	CAZy	*GlycosylTransferase Family 15*	Fungal polysaccharide synthesis	4.54	0.01	*
	15	CAZy	*Glycosyltransferase Family 2*	Fungal polysaccharide synthesis	2.98	0.01	
	16	CAZy	*GlycosylTransferase Family 31*	Fungal polysaccharide synthesis	2.80	0.04	
	17	CAZy	*GlycosylTransferase Family 39*	Fungal polysaccharide synthesis	3.42	0.01	
	18	CAZy	*GlycosylTransferase Family 41*	Fungal polysaccharide synthesis	2.18	0.04	
	19	CAZy	*GlycosylTransferase Family 59*	Fungal polysaccharide synthesis	2.97	0.01	
	20	CAZy	*Polysaccharide Lyase Family 4 Subf 5*	Degradation of polysaccharides	2.13	0.04	
	21	CAZy	*Polysaccharide Lyase Family 7*	Degradation of polysaccharides	2.82	0.01	

Supplementary Figure 8. Frequencies of genes selected from Random Forest (RF) analyses of genomes matching dominant Ascomycota phylotypes (n=13) and genomes representing phylotypes assigned to the non-dominant Ascomycota (n=11). The asterisk highlights those genes that were significantly different in relative frequency (average ± SE), calculated by one-way anova, $p < 0.05$.

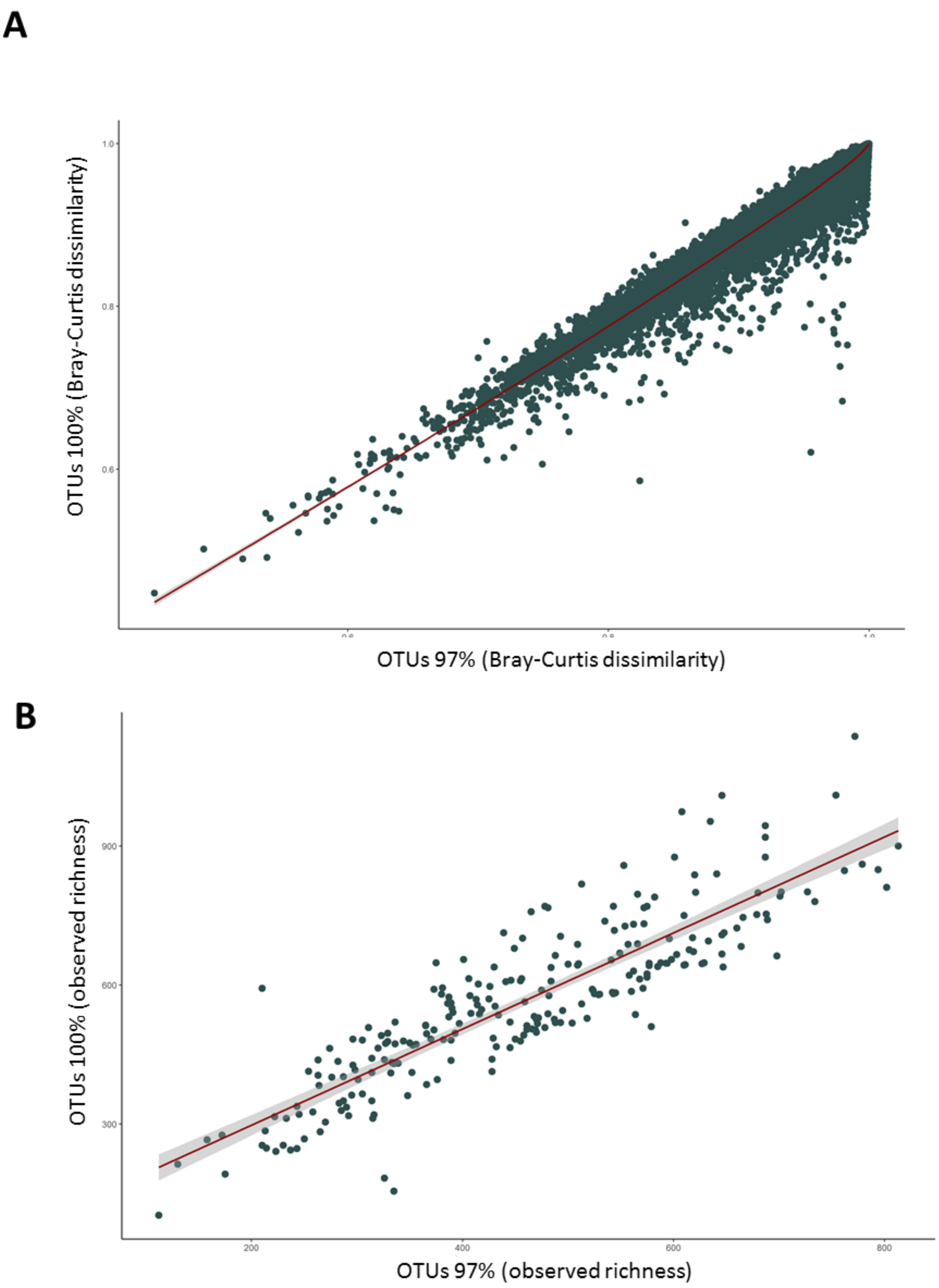

Supplementary Figure 9. Comparison of (A) β (Bray-Curtis dissimilarity index) and (B) observed α diversity for phylotypes clustered at the 97% and 100% similarity cut-off. Shaded areas represent confidence intervals (95%)

Supplementary Note 1

We validated the results of the dominant phylotype identification using data from a recent biogeography study that surveyed the fungal community from soils worldwide [2]. The two datasets are substantially different in terms of methodology, including sampling protocol (top 10 cm of soil here vs. 5 cm), primers used (FITS7/ITS4 here vs. a suite of degenerated ITS3/ITS4), sequencing platform (Illumina MiSeq here vs. pyrosequencing), sequencing depth (12M reads here vs. 2.5M reads), and read lengths (180 bp here vs. 99-409 bp). Additionally, the two datasets differed in the type of biomes surveyed, with the validation dataset having a poor representation of drylands (<1% of the 350 surveyed sites). Therefore, the results of these comparisons should be considered carefully. We used the same approach described in the main text to identify the dominant taxa across the 365 sites of the validation dataset (i.e. taxa that were occurring in at least one third of the samples from a given biome, and were dominant in at least half of the sampled biomes). In the dataset used for validation, we identified 22 dominant phylotypes (vs. the 83 dominant OTUs obtained in our dataset). 45% (n = 10) of the dominant phylotypes from the validation dataset had a matching species hypothesis with the dominant OTUs identified in our dataset, indicating that those phylotypes had at least 97% similarity to each other. Moreover, we found comparable results between the two datasets at coarse taxonomic scales, with the majority of the dominant phylotypes being ascribed to phylum Ascomycota and, to a minor extent, Basiodiomycota and Zygomycota sensu lato. The strongest incongruence was related to Mortierellales, whose members occurred in the dominant community of the validation dataset, but not in our dataset. While members of Mortierellales, including *Mortierella* spp., have been previously retrieved in studies that used the same primer pairs and technologies [3–5], we cannot exclude that the primers used in our survey are not biased against this fungal lineage. Hence, given the methodological differences between the two studies, the discrepancies in number and identity of dominant phylotypes can be ascribed to the differing sequencing depth and overall dissimilarities in sampling and sequencing strategies.

Supplementary Note 2

We also conducted three cross-validations for our predictive maps of the dominant soil fungi distribution. First, we evaluated the correlation between predicted (maps) and observed values for the standardised abundance of dominant taxa of fungi. The two datasets were always positively and significantly correlated using our own global database (Fig. 2; $r = 0.32$-0.61; $P < 0.001$). Subsequently, we re-built the predictive maps using 66% of the data within our database (randomly selected) and the left 33% of data. We found positive significant Spearman correlations between predicted (maps) and observed data (33% of data; $n = 78$) for modules #0 (forest; $r = 0.66$; $P < 0.001$; $n = 78$), #1 (drylands; $r = 0.48$; $P < 0.001$; $n = 78$) and #2 (mesic; $r = 0.48$; $P < 0.001$; $n = 78$). Also, the original maps and the new maps were positively correlated, as follows: modules #0 (forest; $r = 0.46$; $P < 0.001$; $n = 225530$), #1 (dry; $r = 0.51$; $P < 0.001$; $n = 225530$) and #2 (mesic; $r = 0.62$; $P < 0.001$; $n = 225530$). Finally, we used the data from the Biomes of Australian Soil Environments (BASE) soil microbial diversity database (Bissett et al. 2016). BASE is a continental-scale dataset for soil microbes covering a wide variety of bioregions, vegetation and land-use types, allowing us to further cross-validate our results using a large-scale, independent database. First, we extracted the phylotypes from BASE matching (sequence similarity >97%) the top dominant taxa found in our global dataset, resulting in 234 phylotypes accounting for, on average, 9.7% of the total reads. We then focused on the two ecological clusters harbouring the largest number of phylotypes matching our dominant taxa (associated with mesic and dryland systems) for these analyses. Predicted (our global maps) and observed values for the standardised abundance of dominant taxa of fungi were positively and significantly correlated ($r = 0.22$-0.33; $P < 0.001$; $\rho = 0.32$; $P < 0.001$), supporting the validity of our global maps.

Supplementary Table 1

Extended results regarding the mapping of ecological clusters, including correlation coefficients and *p* values for each cluster (module) obtained by Random Forest analyses and semi-partial correlations.

ID	M0	M0	M1	M1	M2	M2
Parameters	Rho	P-value	Rho	P-value	Rho	P-value
Aridity_Index	-0.0819	0.2240	**-0.1502**	**0.0252**	**0.3127**	**0.0000**
Forest	**0.2282**	**0.0006**	-0.0034	0.9600	0.0969	0.1502
Grassland	**0.1619**	**0.0157**	0.0553	0.4121	0.0969	0.1502
MDR	0.0538	0.4252	0.0154	0.8196	**0.1529**	**0.0227**
MAXT	-0.0290	0.6677	-0.0339	0.6154	-0.0844	0.2103
MINT	0.0103	0.8790	-0.0181	0.7890	0.0158	0.8144
UV_Light	**0.1596**	**0.0173**	0.0674	0.3178	0.0495	0.4635
Soil_P	0.0376	0.5775	0.0416	0.5374	0.1092	0.1046
PSEA	-0.0731	0.2779	0.0295	0.6623	0.0902	0.1806
pH	0.1042	0.1215	0.1060	0.1152	-0.0564	0.4032
Clay_silt	0.0611	0.3651	0.0377	0.5766	-0.0443	0.5114
Soil_C	-0.0413	0.5407	-0.0698	0.3004	-0.0444	0.5108
CN	0.0071	0.9156	-0.0285	0.6729	0.0089	0.8952
NPP2003_2015	0.0190	0.7779	-0.0590	0.3813	-0.0065	0.9238

Supplementary Table 2

List of identified dominant fungal phylotypes from soils across the globe from OTUs clustered at the 97% threshold. This list contains information on the taxonomic identity of each phylotype and the most closely related Species Hypothesis.

OTUid	Phylum	Class	Order	Family	Genus	Species Hypothesis
OTU1018	Ascomycota	Ascomycota_unidentified	Ascomycota_unidentified_1	Ascomycota_unidentified_1	Ascomycota_unidentified_1_1	SH189849.06FU
OTU105	Ascomycota	Leotiomycetes	Helotiales	Incertae_sedis_2	Tetracladium	SH216421.06FU
OTU11	Ascomycota	Sordariomycetes	Sordariales	Chaetomiaceae	Humicola	SH234919.06FU
OTU1116	Ascomycota	Lecanoromycetes	Lecanorales	Parmeliaceae		SH206620.06FU
OTU113	Ascomycota	Dothideomycetes	Pleosporales	Pleosporales_unidentified	Pleosporales_unidentified_1	SH196178.06FU
OTU11342	Ascomycota	Ascomycota_unidentified	Ascomycota_unidentified_1	Ascomycota_unidentified_1	Ascomycota_unidentified_1_1	SH233666.06FU
OTU126	Ascomycota	Sordariomycetes	Hypocreales	Hypocreales_unidentified	Hypocreales_unidentified_1	SH224303.06FU
OTU129	Ascomycota	Sordariomycetes	Sordariales	Chaetomiaceae		SH234922.06FU
OTU13	Ascomycota	Ascomycota_unidentified	Ascomycota_unidentified_1	Ascomycota_unidentified_1	Ascomycota_unidentified_1_1	SH219457.06FU
OTU133	Ascomycota	Eurotiomycetes	Chaetothyriales	Herpotrichiellaceae		SH209065.06FU
OTU13559	Ascomycota	Sordariomycetes	Hypocreales	Nectriaceae	Nectriaceae_unidentified	SH217306.06FU
OTU15	Ascomycota	Eurotiomycetes	Chaetothyriales	Herpotrichiellaceae	Herpotrichiellaceae_unidentified	SH209738.06FU
OTU155	Ascomycota	Dothideomycetes	Pleosporales	Sporormiaceae	Sporormiaceae_unidentified	SH190469.06FU
OTU16	Basidiomycota	Tremellomycetes	Tremellales	Incertae_sedis_12	Cryptococcus_1	SH197001.06FU
OTU168	Ascomycota	Dothideomycetes	Pleosporales	Pleosporales_unidentified	Pleosporales_unidentified_1	SH223798.06FU
OTU170	Ascomycota	Sordariomycetes	Hypocreales	Ophiocordycipitaceae	Purpureocillium	SH224001.06FU
OTU186	Ascomycota	Dothideomycetes	Pleosporales	Sporormiaceae	Preussia	SH190474.06FU
OTU2	Ascomycota	Sordariomycetes	Hypocreales	Nectriaceae	Fusarium	SH217297.06FU
OTU20	Ascomycota	Sordariomycetes	Hypocreales	Hypocreaceae	Trichoderma	SH240600.06FU
OTU2009	Ascomycota	Dothideomycetes	Pleosporales	Pleosporaceae	Alternaria	SH224792.06FU
OTU21	Ascomycota	Dothideomycetes	Incertae_sedis_8	Pseudeurotiaceae	Pseudogymnoascus	SH236509.06FU
OTU2182	Ascomycota	Eurotiomycetes	Eurotiales	Trichocomaceae	Penicillium	SH193628.06FU
OTU23	Ascomycota	Sordariomycetes	Sordariales	Sordariaceae	Sordariaceae_unidentified	SH213056.06FU
OTU2322	Ascomycota	Ascomycota_unidentified	Ascomycota_unidentified_1	Ascomycota_unidentified_1		SH234925.06FU
OTU242	Ascomycota	Dothideomycetes	Pleosporales	Pleosporaceae	Bipolaris	SH224794.06FU
OTU246	Ascomycota	Dothideomycetes	Dothideales	Dothioraceae	Aureobasidium	SH206629.06FU
OTU259	Ascomycota	Sordariomycetes	Sordariales	Chaetomiaceae		SH234919.06FU
OTU261	Ascomycota	Sordariomycetes	Hypocreales	Hypocreales_unidentified	Hypocreales_unidentified_1	SH217356.06FU
OTU27	Ascomycota	Ascomycota_unidentified	Ascomycota_unidentified_1	Ascomycota_unidentified_1		SH236035.06FU
OTU276	Ascomycota	Eurotiomycetes	Chaetothyriales	Chaetothyriales_unidentified	Chaetothyriales_unidentified_1	SH209756.06FU
OTU280	Ascomycota	Eurotiomycetes	Chaetothyriales	Herpotrichiellaceae		SH225357.06FU
OTU288	Ascomycota	Sordariomycetes	Coniochaetales	Coniochaetaceae	Coniochaeta	SH226462.06FU
OTU296	Ascomycota	Dothideomycetes	Pleosporales	Pleosporales_unidentified		SH240086.06FU
OTU3	Ascomycota	Ascomycota_unidentified	Ascomycota_unidentified_1	Ascomycota_unidentified_1	Ascomycota_unidentified_1_1	SH197541.06FU
OTU30	Ascomycota	Sordariomycetes	Sordariomycetes_unidentified	Sordariomycetes_unidentified_1	Sordariomycetes_unidentified_1	SH235275.06FU

OTU301	Ascomycota	Dothideomycetes	Pleosporales	Pleosporales_unidentified	Pleosporales_unidentified_1	SH217121.06FU
OTU330	Ascomycota	Eurotiomycetes	Chaetothyriales	Herpotrichiellaceae	Capronia	SH209735.06FU
OTU340	Ascomycota	Sordariomycetes	Hypocreales	Bionectriaceae	Clonostachys	SH236629.06FU
OTU355	Basidiomycota	Tremellomycetes	Tremellales	Incertae_sedis_12		SH197113.06FU
OTU356	Ascomycota	Sordariomycetes	Sordariales	Lasiosphaeriaceae	Podospora	SH222042.06FU
OTU4	Ascomycota	Dothideomycetes	Pleosporales	Pleosporaceae	Pleosporaceae_unidentified	SH224797.06FU
OTU40	Ascomycota	Eurotiomycetes	Chaetothyriales	Chaetothyriales_unidentified	Chaetothyriales_unidentified_1	SH219002.06FU
OTU435	Ascomycota	Dothideomycetes	Pleosporales	Pleosporales_unidentified	Pleosporales_unidentified_1	SH237285.06FU
OTU44	Ascomycota	Leotiomycetes	Helotiales	Vibrisseaceae	Myxocephala	SH235575.06FU
OTU45	Ascomycota	Sordariomycetes	Coniochaetales	Coniochaetaceae	Lecythophora	SH235858.06FU
OTU537	Ascomycota	Dothideomycetes	Pleosporales	Montagnulaceae	Paraconiothyrium	SH224124.06FU
OTU541	Ascomycota	Sordariomycetes	Hypocreales	Cordycipitaceae	Lecanicillium	SH196289.06FU
OTU577	Ascomycota	Eurotiomycetes	Eurotiales	Trichocomaceae	Penicillium	SH231334.06FU
OTU58	Ascomycota	Eurotiomycetes	Chaetothyriales	Herpotrichiellaceae	Exophiala	SH230191.06FU
OTU59	Ascomycota	Leotiomycetes	Incertae_sedis	Incertae_sedis_1	Leohumicola	SH209174.06FU
OTU6	Ascomycota	Dothideomycetes	Pleosporales	Pleosporaceae	Alternaria	SH224789.06FU
OTU61	Ascomycota	Sordariomycetes	Sordariales	Chaetomiaceae	Chaetomiaceae_unidentified	SH234920.06FU
OTU64	Ascomycota	Eurotiomycetes	Eurotiales	Trichocomaceae	Talaromyces	SH225847.06FU
OTU67	Ascomycota	Sordariomycetes	Sordariales	Chaetomiaceae		SH234934.06FU
OTU6748	Ascomycota	Dothideomycetes	Pleosporales	Incertae_sedis_13		SH202145.06FU
OTU7	Ascomycota	Ascomycota_unidentified	Ascomycota_unidentified_1	Ascomycota_unidentified_1	Ascomycota_unidentified_1_1	SH217298.06FU
OTU73	Ascomycota	Sordariomycetes	Hypocreales	Hypocreaceae	Trichoderma	SH222750.06FU
OTU747	Ascomycota	Eurotiomycetes	Chaetothyriales	Herpotrichiellaceae	Cladophialophora	SH225359.06FU
OTU7611	Ascomycota	Sordariomycetes	Sordariales	Lasiosphaeriaceae	Podospora	SH222041.06FU
OTU8	Ascomycota	Ascomycota_unidentified	Ascomycota_unidentified_1	Ascomycota_unidentified_1		SH241083.06FU
OTU807	Ascomycota	Dothideomycetes	Pleosporales	Pleosporaceae		SH197545.06FU
OTU82	Ascomycota	Ascomycota_unidentified	Ascomycota_unidentified_1	Ascomycota_unidentified_1	Ascomycota_unidentified_1_1	SH195163.06FU
OTU9	Ascomycota	Dothideomycetes	Capnodiales	Davidiellaceae	Davidiella	SH196750.06FU

Supplementary References:

1. Oksanen, A. J. *et al.* Package ‘ vegan ’. (2013).
2. Tedersoo, L. *et al.* Global diversity and geography of soil fungi. *Science (80-.).* 346, (2014).
3. Nallanchakravarthula, S., Mahmood, S., Alström, S. & Finlay, R. D. Influence of Soil Type, Cultivar and Verticillium dahliae on the Structure of the Root and Rhizosphere Soil Fungal Microbiome of Strawberry. *PLoS One* 9, e111455 (2014).
4. Gkarmiri, K. *et al.* Identifying the Active Microbiome Associated with Roots and Rhizosphere Soil of Oilseed Rape. *Appl. Environ. Microbiol.* 83, AEM.01938-17 (2017).
5. Hamonts, K. *et al.* Field study reveals core plant microbiota and relative importance of their drivers. *Environ. Microbiol.* 20, 124–140 (2018).
6. Nei, M. & Kumar, S. *Molecular evolution and phylogenetics*. (2000).
7. Kumar, S., Stecher, G. & Tamura, K. MEGA7: Molecular Evolutionary Genetics Analysis Version 7.0 for Bigger Datasets. *Mol. Biol. Evol.* 33, 1870–1874 (2016).
8. Letunic, I. & Bork, P. Interactive tree of life (iTOL) v3: an online tool for the display and annotation of phylogenetic and other trees. *Nucleic Acids Res.* 44, W242–W245 (2016).
