# Supplementary material for: A few Ascomycota taxa dominate soil fungal communities worldwide
Source: Nat Commun. 2019 May 30;10:2369. doi: 10.1038/s41467-019-10373-z (PMC6542806; doi:10.1038/s41467-019-10373-z)
Supplement: Supplementary file 3 — Reporting Summary [file 41467_2019_10373_MOESM3_ESM.pdf]

natureresearch

Corresponding author(s): Eleonora Egidi, Manuel Delgado-Baquerizo, Brajesh Singh

# Reporting Summary

Nature Research wishes to improve the reproducibility of the work that we publish. This form provides structure for consistency and transparency in reporting. For further information on Nature Research policies, see Authors & Referees and the Editorial Policy Checklist.

## Statistical parameters

When statistical analyses are reported, confirm that the following items are present in the relevant location (e.g. figure legend, table legend, main text, or Methods section).

| n/a | Confirmed | |
|---|---|---|
| ☐ | ☒ | The exact sample size ($n$) for each experimental group/condition, given as a discrete number and unit of measurement |
| ☒ | ☐ | An indication of whether measurements were taken from distinct samples or whether the same sample was measured repeatedly |
| ☐ | ☒ | The statistical test(s) used AND whether they are one- or two-sided<br>*Only common tests should be described solely by name; describe more complex techniques in the Methods section.* |
| ☐ | ☒ | A description of all covariates tested |
| ☐ | ☒ | A description of any assumptions or corrections, such as tests of normality and adjustment for multiple comparisons |
| ☐ | ☒ | A full description of the statistics including central tendency (e.g. means) or other basic estimates (e.g. regression coefficient) AND variation (e.g. standard deviation) or associated estimates of uncertainty (e.g. confidence intervals) |
| ☐ | ☒ | For null hypothesis testing, the test statistic (e.g. $F$, $t$, $r$) with confidence intervals, effect sizes, degrees of freedom and $P$ value noted<br>*Give P values as exact values whenever suitable.* |
| ☒ | ☐ | For Bayesian analysis, information on the choice of priors and Markov chain Monte Carlo settings |
| ☒ | ☐ | For hierarchical and complex designs, identification of the appropriate level for tests and full reporting of outcomes |
| ☐ | ☒ | Estimates of effect sizes (e.g. Cohen's $d$, Pearson's $r$), indicating how they were calculated |
| ☐ | ☒ | Clearly defined error bars<br>*State explicitly what error bars represent (e.g. SD, SE, CI)* |

*Our web collection on statistics for biologists may be useful.*

## Software and code

Policy information about availability of computer code

| | |
|---|---|
| Data collection | Gene predictions: Mycocosm database, Resistance Gene Identifier (RGI) algorithm and Comprehensive Antibiotic Resistance Database (CARD) |
| Data analysis | Sequencing data processing: USEARCH (v10), UNITE fungal database (v7.2)<br>Phylogenetic tree reconstruction: Mega (v7), iTOL (v3)<br>Habitat preferences: gephi (v0.9.2), ppcor r package<br>Gene analysis: rfPermute r package, mvabund r package |

For manuscripts utilizing custom algorithms or software that are central to the research but not yet described in published literature, software must be made available to editors/reviewers upon request. We strongly encourage code deposition in a community repository (e.g. GitHub). See the Nature Research guidelines for submitting code & software for further information.



April 2018

## Data

Policy information about availability of data

All manuscripts must include a data availability statement. This statement should provide the following information, where applicable:

- Accession codes, unique identifiers, or web links for publicly available datasets
- A list of figures that have associated raw data
- A description of any restrictions on data availability

The raw reads are available at https://figshare.com/s/9772d31625426d907782 (DOI: 10.6084/m9.figshare.5923876). All other relevant data is available upon request.

# Field-specific reporting

Please select the best fit for your research. If you are not sure, read the appropriate sections before making your selection.

☐ Life sciences ☐ Behavioural & social sciences ☒ Ecological, evolutionary & environmental sciences

For a reference copy of the document with all sections, see nature.com/authors/policies/ReportingSummary-flat.pdf

# Ecological, evolutionary & environmental sciences study design

All studies must disclose on these points even when the disclosure is negative.

| | |
|---|---|
| Study description | In this work, we surveyed 235 sites collected across 18 countries, covering nine biomes (temperate, tropical and dry forests, cold, temperate, tropical and arid grasslands, shrubland, boreal) of the globe to investigate identity, occurrence and ecology of dominant fungal phylotypes in soil. We identified the dominant fungal phylotypes (i.e., abundant and frequent fungi) and used a suite of statistical approaches to characterise their distribution and habitat preferences in relation to 14 environmental predictors: climate variables (Aridity Index, minimum and maximum temperature, precipitation seasonality and mean diurnal temperature range – MDR), UV radiation, net primary productivity (NDVI index, 2003-2015 period), soil properties (texture [% of clay + silt], soil pH, total C, N and P concentrations and C: N ratio) and dominant ecosystem types in our dataset (forest and grasslands). We then mapped their expected geographical distribution and abundance. Additionally, we used whole-genome comparisons (n=33 fungal genomes) to assess the importance of functional attributes in explaining the observed patterns of fungal dominance. |
| Research sample | We used soil samples, from whom genomic DNA was extracted |
| Sampling strategy | At each site, a composite soil sample (top ~7.5cm depth) was collected under the most common vegetation. After field collection, each soil sample was separated into two sub-samples - one subsample was immediately frozen at -20 ºC for molecular analyses while the other sub-sample was air-dried for chemical analyses. |
| Data collection | M.D.-B., F.T.M., D.J.E., and B.K.S. collected the soil samples. The coordinates of each site were recorded in situ with a portable GPS, and the ecosystem type (grassland, shrubland, or forest) of each location recorded. Soil physico-chemical and climatic variables were obtained from Delgado-Baquerizo,et al., Science 359.6373 (2018): 320-325. Fungal sequence data was determined by sequencing the Internal Transcribed Spacer (ITS) region 2 with primers FITS7/ITS4 on an Illumina MiSeq machine. Genomes were retrieved from the Mycocosm database, and annotated using Resistance Gene Identifier (RGI) algorithm and Comprehensive Antibiotic Resistance Database (CARD) |
| Timing and spatial scale | Soil sample collection took place between 2003 and 2015, and encompassed 6 continents (Africa (n=18), Europe (n=31), Asia (n=10), Australia (n=85), North (n=78) and South (n=15) America), covering nine biomes (temperate (n=27), tropical (n=7) and dry forests (n=60), cold (n=18), temperate (n=27), tropical (n=7) and arid (n=42) grasslands, shrubland (n=15), boreal (n=4)) across the globe. |
| Data exclusions | no data were excluded from the analysis |
| Reproducibility | To avoid biases associated with having multiple laboratories analyzing soils from different sites, and to facilitate the comparison of results between them, all dried soil samples were shipped to the Universidad Rey Juan Carlos (Spain) for laboratory analyses, while the extracted DNA samples were frozen and shipped to the Next Generation Genome Sequencing Facility of the University of Western Sydney (Australia) for fungal ITS sequencing. We validated the results of the dominant phylotype identification using data from a recent biogeography study that surveyed the fungal community from soils worldwide (Tedersoo et al. Science 346.6213 (2014): 1256688). We implemented the same approach used in our dataset to identify the dominant taxa across the 365 sites of the validation dataset. We found comparable results between the two datasets at coarse taxonomic scales, with 45% of the dominant phylotypes from the validation dataset having a matching (97% similarity) species hypothesis with the dominant OTUs identified in our dataset |
| Randomization | DNA samples were randomly processed for sequencing |
| Blinding | Blinding was not relevant for this study |

Did the study involve field work? ☒ Yes ☐ No

## Field work, collection and transport

| | |
|---|---|
| Field conditions | This study includes locations spanning a wide range of mean temperatures (-11.4 to 26.5 ºC) and precipitation (67-3085mm) regimes |
| Location | This study included locations spanning a wide range of latitudes (from -54.972S to 68.633N), and longitudes (155.26W to 151.14E) |
| Access and import/export | Permits were needed to import DNA to Australia. |
| Disturbance | Not applicable |

# Reporting for specific materials, systems and methods

### Materials & experimental systems

| n/a | Involved in the study |
|---|---|
| ☒ | ☐ Unique biological materials |
| ☒ | ☐ Antibodies |
| ☒ | ☐ Eukaryotic cell lines |
| ☒ | ☐ Palaeontology |
| ☒ | ☐ Animals and other organisms |
| ☒ | ☐ Human research participants |

### Methods

| n/a | Involved in the study |
|---|---|
| ☒ | ☐ ChIP-seq |
| ☒ | ☐ Flow cytometry |
| ☒ | ☐ MRI-based neuroimaging |
