# Supplementary material for: A few Ascomycota taxa dominate soil fungal communities worldwide
Source: Nat Commun. 2019 May 30;10:2369. doi: 10.1038/s41467-019-10373-z (PMC6542806; doi:10.1038/s41467-019-10373-z)
Supplement: Supplementary file 4 — Description of Additional Supplementary Files [file 41467_2019_10373_MOESM4_ESM.pdf]

Description of Additional Supplementary Files

File Name: Supplementary Data 1
Description: List of identified dominant fungal phylotypes from soils across the globe. This list contains the phylotype sequence, as well as information on the taxonomic identity of each phylotype, the ecological cluster it was assigned to, and the most closely related genbank sequence and Species Hypothesis.

File Name: Supplementary Data 2
Description: List of whole genomes used in this study, including identification number and reference to the study where they have been published.
